# Efficacy and Safety of Open-Conjunctiva Ab Externo 63 µm vs. 45 µm XEN^®^ Gel Stent in Glaucoma Surgery: One-Year Follow-Up

**DOI:** 10.3390/jcm14103545

**Published:** 2025-05-19

**Authors:** Yann Bertolani, Jaume Rigo-Quera, Laura Sánchez-Vela, Olivia Pujol-Carreras, Manuel Amilburu, Antonio Dou, Marta Castany

**Affiliations:** Department of Ophthalmology, Vall d’Hebron University Hospital, 08035 Barcelona, Spain

**Keywords:** XEN^®^ 63 µm, XEN^®^ 45 µm, minimally invasive glaucoma surgery, minimally invasive bleb surgery, intraocular surgery, open-conjunctiva approach, survival analysis

## Abstract

**Background**: To compare the efficacy and safety of the XEN^®^ 63 µm and 45 µm devices with the ab externo open conjunctiva with a 30G needle approach. **Methods**: A retrospective, non-randomized and single-center study was conducted. Consecutive eyes undergoing a XEN^®^ 63 µm implant were compared with a matched cohort of cases with a XEN^®^ 45 µm implant. Standalone and combined procedures with phacoemulsification were included. **Results**: A total of 28 XEN^®^ 45 µm and 28 XEN^®^ 63 µm were included. Complete surgical success was achieved in 17 cases (60.7%) in the 45 µm group and in 20 cases (71.4%) in the 63 µm group, with no statistical differences. One year after the surgery, the mean IOP was 13.8 ± 3.3 mmHg for the 45 µm group and 12.4 ± 4.2 mmHg for the 63 µm group (*p*-value > 0.05). Likewise, the use of glaucoma medication was lowered in the 63 µm device (0.32 ± 0.87) compared to the 45 µm device (0.39 ± 0.86), with no statistical significance. Postoperative hypotony was more frequent in the 63 µm device (39.3%) than in the 45 µm group (28.6%), with no statistical differences. However, hypotony-associated complications (including choroidal detachment, hypotony keratopathy, and hypotony maculopathy) were significantly higher in the 63 µm group (*p* = 0.011). **Conclusions:** Although the XEN^®^ 63 µm may offer a greater IOP-lowering effect with better complete surgical success, no significant differences were detected compared to the 45 µm device. Hypotony-related complications were higher in the XEN 63 µm, although most of them resolved with conservative management.

## 1. Introduction

Glaucoma is a chronic and progressive optic neuropathy characterized by the loss of ganglion cells and associated visual loss [1]. Thus, early diagnosis and treatment are crucial to prevent further progression and to limit the socioeconomic burden of the disease. Lowering intraocular pressure (IOP) is the major modifiable factor to reduce the disease progression, and it may be achieved through pharmacological treatment, laser, or surgery [2]. In recent years, minimally invasive glaucoma surgery (MIGS) devices have gained popularity as an alternative to conventional filtering surgery, as they may offer a safer profile, limiting postoperative hypotony with comparable efficacy [3,4]. The XEN^®^ Gel Stent implant (AbbVie Inc., Chicago, IL, USA), derived from porcine gelatin, decreases IOP by facilitating the drainage of aqueous humor from the anterior chamber to the subconjunctival space [5]. It is a non-valved device based on the Hagen–Poiseuille principle, designed to reduce the incidence of early postoperative hypotony and associated complications [6]. As its mechanism depends on the formation of a filtration bleb, some authors have advocated for classifying the XEN implant as a minimally invasive bleb surgery (MIBS) device [7].

Currently, two calibers of XEN^®^ devices are available based on the lumen diameter: the XEN^®^ 45 µm and the XEN^®^ 63 µm. Several studies have reported the efficacy and safety of the XEN^®^ 45 µm device in the surgical treatment of glaucoma [8,9]. As suggested by the manufacturer, the 63 µm may offer a greater IOP reduction because of the increased diameter compared to the 45 µm device, while maintaining an adequate safety profile. The XEN^®^ was initially designed to be implanted with an ab interno closed-conjunctiva (AIC) approach [10]. In recent years the off-label ab externo approach (with both closed and open conjunctiva) has been described, demonstrating its safety and efficacy in clinical practice [11,12,13,14]. A limited number of studies have been conducted comparing the efficacy and safety of the XEN^®^ 45 µm and the XEN^®^ 63 µm [15,16]. However, to our knowledge, this is the first study conducted to compare the outcomes between both devices with the ab externo open-conjunctiva (AEO) approach. We believe this comparative evaluation of the two devices will provide relevant information for the optimization and individualization of the surgical approach to glaucoma.

## 2. Materials and Methods

### 2.1. Study Design and Ethics Statement

This is a retrospective, non-randomized, single-center cohort study of consecutive patients who underwent either standalone or combined with phacoemulsification XEN^®^ 63 µm Gel Stent implantation. The results were compared with a matched cohort of patients with a standalone or combined with phacoemulsification XEN^®^ 45 µm Gel Stent surgery. All procedures were performed at the Glaucoma Department of the Vall d’Hebron Hospital, with a minimum follow-up of one year. The approval from our Institutional Review Board (CEIm of the Vall d’Hebron University Hospital) was obtained for the review of the patients’ clinical records (Protocol PS(AG)019/2024(6350), 8 November 2024). This research complies with the Good Clinical Practice/International Council for Harmonization Guidelines and adheres to the Declaration of Helsinki. 

### 2.2. Study Participants and Data Collection

Patients older than 18 years of age with a diagnosis of primary open-angle, pseudoexfoliative, pigment dispersion, normal-tension, steroid-induced, uveitic, juvenile, or combined-mechanism glaucoma who underwent XEN^®^ 63 µm or XEN^®^ 45 µm were included. Standalone or combined procedures with phacoemulsification were recorded. Patients with primary or secondary angle closure, neovascular, and iridocorneal endothelial syndrome-associated glaucoma were excluded from the present study. Moreover, patients with any surgical procedure (including cataract surgery) 6 months prior to surgery, vitreous in the anterior chamber, the presence of intraocular silicone oil, or allergy to any medication required for implantation or any of the device components were not included. Surgery was decided in the following scenarios: unmet target IOP despite maximum tolerated topical medical therapy, intolerance to medical therapy, and documented structural glaucomatous progression, with or without associated functional deterioration.

The electronic medical records were accessed to collect the data. Recorded baseline characteristics included age, sex, laterality, preoperative visual acuity (logMAR), preoperative IOP using Goldmann applanation tonometry, number of glaucoma medications, glaucoma type and disease staging, previous selective laser trabeculoplasty (SLT), previous glaucoma surgeries, other previous surgeries, axial length, and mean deviation (MD) in SITA fast 24-2 program visual field (VF) (Carl Zeiss Meditec, Inc., Dublin, CA, USA). All patients underwent a thorough ophthalmological examination, including a review of the medical and ophthalmological history, IOP measurements, gonioscopy, and glaucoma staging through optic nerve–head optical coherence tomography (ONH-OCT) (Cirrus^®^ OCT 500, Zeiss, Oberkochen, Germany) and 24.2 VF (Humphrey Field Analyzer 3^®^, Zeiss, Oberkochen, Germany). The postoperative visit regime (1 day, 1 week, and 1, 2, 3, 6, and 12 months) included ophthalmic examination with IOP measurements. The requirements in needlings, ocular hypotensive medication, and other surgical procedures were recorded. Anterior segment optical coherence tomography (AS-OCT) (Anterion^®^, Heidelberg, Germany or Triton^®^, Topcon, Tokyo, Japan) to assess the bleb morphology and filtration was performed at the ophthalmologist’s discretion. ONH-OCT and 24.2° VF were performed to assess functional or structural progression at 6 months and 1 year after the surgery. Intraoperative and postoperative adverse events, the need for surgical bleb revision, and additional required glaucoma surgeries were recorded. The procedures were performed by different glaucoma surgeons with expertise in MIGS and MIBS.

### 2.3. Surgical Technique

**Figure 1:**
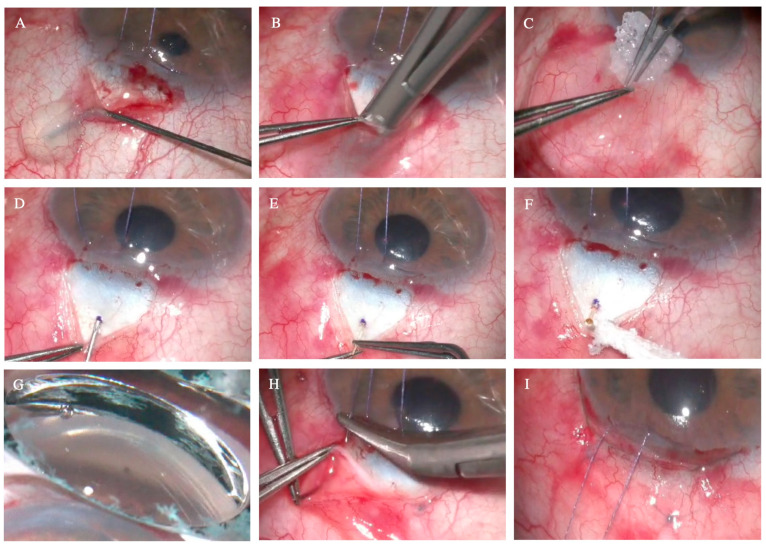
Surgical technique for the 30G needle-mediated open-conjunctiva ab externo XEN^®^ 45 and 63 µm implants. (**A**) Instillation of sub-Tenon’s anesthetic (2% lidocaine with epinephrine) after initial peritomy with Vanna’s scissors. (**B**) Dissection of conjunctiva and the Tenon’s capsule with Wescott scissors. (**C**) Application of MMC 0.02% on the scleral bed. (**D**) Marking at 2 mm from the surgical limbus and creation of the scleral tract with a 30G needle. (**E**) Manual insertion of the XEN^®^ implant using non-toothed forceps. (**F**) Verification of correct tubular filtration at the distal segment of the XEN. (**G**) Verification of the correct implantation of the device with a gonioscopy lens. (**H**) Tenon’s capsule closure with interrupted Vicryl 7.0 sutures. (**I**) Closure of the conjunctiva with interrupted 10.0 Nylon sutures.

The XEN^®^ Gel Stent was implanted with an AEO approach, using the same surgical technique for both the 45 µm and the 63 µm devices, as depicted in Figure 1. Retrobulbar anesthesia was performed by instilling 2 mL of a combination of 2% mepivacaine and 0.5% bupivacaine, except in eyes with severe glaucoma, with high risk of wipe-out, and at the surgeon’s discretion. In patients undergoing combined surgery, phacoemulsification with intraocular lens implantation was performed first, followed by closure of the main incision with a 10.0 Nylon suture. A superior and temporal corneal traction suture with 7.0 Vicryl was placed to optimize exposure of the conjunctiva. The XEN was implanted in the upper temporal area, avoiding the superior area to prevent conjunctival scarring if secondary filtering surgery was required. In cases with previous glaucoma surgery, the XEN placement was displaced to the temporal sector rather than to the nasal area due to its greater risk of generating postoperative dysesthetics blebs. After an initial peritomy with Vanna’s scissors, sub-Tenon’s anesthesia (2% lidocaine combined with 2.5% epinephrine) was instilled with a 30G canula. Subsequently, a superior fornix-based conjunctival flap was created, followed by a blunt dissection of the Tenon’s capsule and conjunctiva with Westcott’s scissors. Diathermy was applied to the scleral bed to ensure appropriate hemostasis, and 0.02% mitomycin C (MMC) soaked up in Spongostan^TM^ was applied for 2 min, followed by gentle washing with a balanced salt solution (BSS). A scleral tract was made using a bent 30G needle 2 mm away from the surgical limbus. The XEN implant was removed from its injector and manually positioned with atraumatic forceps through the scleral tract. When handling the device, caution was taken, avoiding exposure to BSS to preserve its rigidity and to facilitate its insertion. If the XEN had been in contact with the BSS, the hydrated device may have been difficult to implant through a 30G scleral tract. In those cases, an alternative tract with a 27G needle was created for the manual insertion of the XEN. Adequate tubular filtration and the absence of peritubular filtration were assessed. A gonioscopy lens was used during the surgical procedure to confirm the appropriate positioning of the device in the anterior chamber, ensuring a length of 3 mm under the conjunctiva, 2 mm in the intrascleral course, and 1 mm in the anterior chamber (the 3–2–1 rule).

Healaflow^®^ (MedicalMix, Barcelona, Spain) or Ologen^®^ (Equipsa, Madrid, Spain) filtration bleb modulators were positioned on the scleral bed around the XEN implant as a space-occupying agent to separate the XEN lumen from the Tenon’s capsule. Initially, the preferred modulator was Ologen^®^, until supply issues arose, after which Healaflow^®^ began to be used. Finally, the Tenon’s capsule and conjunctiva were closed in planes, using Vicryl 7.0 and 10.0 Nylon sutures, respectively. Although the Tenon’s capsule and the conjunctiva may be sutured together, we considered that closing in planes would avoid the posterior displacement of the Tenon, achieving better postoperative outcomes. At the end of the procedure, intracameral 0.1 mL cefuroxime was instilled in the anterior chamber.

### 2.4. Postoperative Regime, Procedures, and Care

Patients were instructed to discontinue the use of systemic and topical hypotensive medication at the time of surgery. Postoperative treatment included topical ciprofloxacin every 6 h for one week and topical phosphate dexamethasone every 2 h for three weeks with further tapering. Cycloplegic and atropine were used in cases of hypotony-associated complications such as hypothalamia, choroidal detachment or hypotonic maculopathy. Patients were evaluated postoperatively at the following time points: one day, one week, one month, two months, three months, six months, and one year after the surgery. The visiting scheme was modified depending on the postoperative evolution, the presence of complications, and at the discretion of the surgeon. Secondary needling was performed in cases with bleb fibrosis and Tenon’s cysts, assessed by AS-OCT with a 30G hypodermic needle in the subconjunctival space, releasing the episcleral adhesions below and above the device. The use of MMC 0.01% was associated with the procedure at the ophthalmologist’s discretion.

### 2.5. Surgical Success and Outcomes

The objective of this study was to assess differences in efficacy and safety between both devices. Thus, the primary outcome of this study was the complete surgical success rate, defined as an IOP ≤ 18 mmHg, with a reduction of ≥20% from preoperative values, with no use of hypotensive medication. Qualified success included patients achieving an IOP of ≤18 mmHg and a ≥20% reduction from baseline while using glaucoma medications. Failure was defined as functional or structural disease progression, IOP > 18 mmHg, loss of visual acuity secondary to hypotony related complications or glaucoma progression 1 year after the surgery, the need for a bleb surgical revision due to an unmet IOP objective, or the need for additional glaucoma surgeries to achieve IOP control. The secondary outcomes of this study included the mean postoperative IOP and the mean number of required topical hypotensive medications 1 year after the procedure. Furthermore, additional secondary outcomes comprised the need for secondary needling, surgical reintervention, and both early and late postoperative complications.

### 2.6. Statistical Analysis

Statistical analyses were conducted with IBM SPSS ver. 30.0 (IBM Corp, New York, NY, USA). The normality of the data distribution was assessed with the Kolmogorov–Smirnov test. Descriptive analyses of baseline characteristics and follow-up measurements were performed. Categorical variables are presented as absolute frequencies and percentages. Continuous variables are described as mean and standard deviation (SD) if they follow a normal distribution and as median and interquartile range (IQR) if they do not follow a normal distribution. A Student’s *t*-test was used to assess statistical significance for mean value changes from baseline in both groups of treatments. In cases of quantitative variables with no normal distribution, the Mann-Whitney U-test and the Wilcoxon signed rank test were calculated to assess statistical significance. When comparing categorical variables, the Chi-square or the Fisher’s exact test were evaluated. Kaplan–Meier survival analyses were used to evaluate both complete and qualified surgical success, comparing the surgical outcomes between the two devices. Moreover, multivariable Cox regression models with hazard ratio (HR) were adjusted for baseline IOP and the use of bleb space modulators. All tests were two-tailed, and a *p* < 0.05 was considered statistically significant.

## 3. Results

### 3.1. Baseline Characteristics

The study included 28 eyes from 27 patients who had undergone AEO XEN^®^ 63 µm implantation and 28 eyes from 28 patients with an AEO XEN^®^ 45 µm implant. The demographic characteristics were similar between both treatment groups, with no significant differences. Preoperative clinical and demographic data are presented in Table 1.

At baseline, the XEN^®^ 63 µm group had a mean IOP of 23.9 ± 2.5 mmHg with a mean of 2.6 ± 0.8 glaucoma medications compared with a mean IOP of 23.1 ± 2.2 mmHg with a mean of 2.5 ± 1.1 glaucoma medications in the 45 µm group. There were no statistical differences regarding the type of glaucoma distribution, the preoperative lens status, disease staging, and previous glaucoma or ophthalmological procedures between both groups. Combined surgery was performed in 9 (32.1%) and 11 (39.3%) cases in the 63 µm and 45 µm groups, respectively, with no statistical differences.

### 3.2. Efficacy

**Figure 2:**
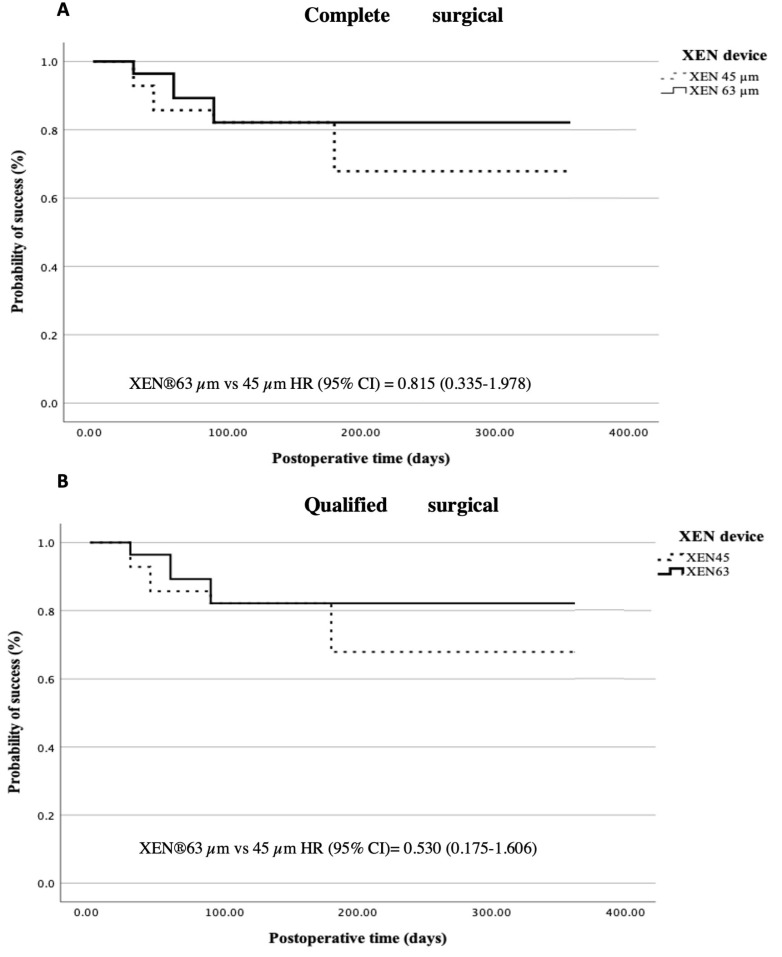
Kaplan–Meier analysis for complete (**A**) and qualified (**B**) surgical success. Hazard radio (HR) adjusted by baseline IOP and use of bleb space modulators.

The Kaplan–Meier survival curves for complete and qualified surgical success, adjusted for baseline IOP and the use of bleb space modulators, are presented in Figure 2A,B). Complete surgical success was achieved in 17 cases (60.7%) in the 45 µm group and in 20 cases (71.4%) in the 63 µm group. Additionally, qualified surgical success was accomplished in 19 eyes (67.9%) in the AEO XEN^®^ 45 µm group and in 23 eyes (82.1%) in the XEN^®^ 63 µm group. For multivariable Cox regression, both complete (HR (95% CI) = 0.815 (0.335–1.978)) and qualified (HR (95% CI) = 0.530 (0.175–1.606)) survival failure rates were lower in the XEN^®^ 63 µm device, with no statistical significance.

**Figure 3:**
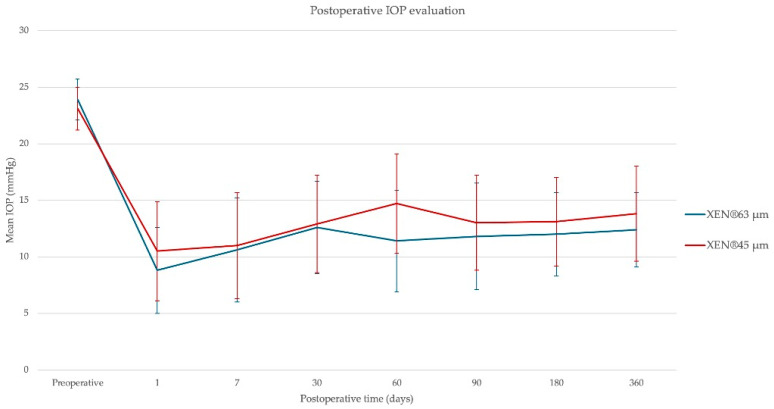
Postoperative mean IOP evolution in the XEN^®^ 45 µm and the XEN^®^ 63 µm. The error bars indicate standard deviations.

The mean IOP and the mean number of glaucoma medications were significantly lowered 1 year after the surgery in both treatment groups. Although the trend suggested a lower mean postoperative IOP in the 63 µm group, no significant differences were observed between the two treatment groups at the different follow-up times (24 h, 1 week, and 1, 2, 3, 6, and 12 months) (Figure 3). One year after the surgery, the mean IOP was 13.8 ± 3.3 mmHg for the 45 µm group and 12.4 ± 4.2 mmHg for the 63 µm group (T-Student, *p* = 0.175). No statistical differences were found based on the use or not of bleb space modulators in mean IOP 1 year after the procedure.

Likewise, both devices achieved a significant reduction in the use of glaucoma medications to achieve an optimal IOP control. Although the requirements for antihypertensive drugs were lower in the 63 µm device (0.32 ± 0.87) compared to the 45 µm device (0.39 ± 0.86), these differences did not reach statistical significance (Mann–Whitney U-test, *p* = 0.264). The incidence of secondary needling was similar in both groups of treatments (5 and 6 eyes in the 45 µm and the 63 µm, respectively) (Pearson Chi-square, *p* = 0.737). Surgical bleb revision due to fibrosis was conducted in two XEN^®^ 45 µm eyes. Additionally, three cases (10.7%) in the 45 µm group and two cases (7.1%) in the 63 µm group required a secondary glaucoma procedure to achieve optimal IOP control. As secondary procedures, non-penetrating deep sclerectomy (NPDS) was performed in four cases and trabeculectomy (TBT) in the remaining one.

### 3.3. Safety

**Figure 4:**
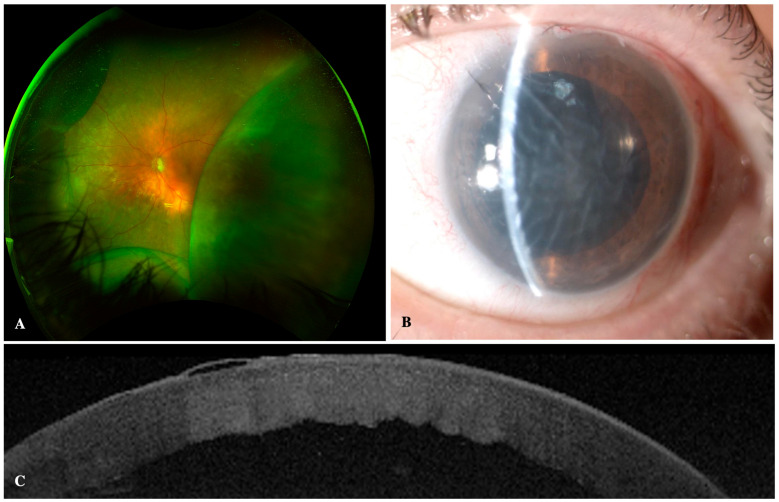
Ab externo open-conjunctiva XEN^®^-associated complications. (**A**) Multifocal serous choroidal detachments after XEN^®^ 63 µm implantation. (**B**) Urretz–Zavalia syndrome, toxic anterior segment syndrome, and corneal edema after XEN^®^ 45 µm implantation. (**C**) Anterior segment optical coherence tomography (Triton^®^, Topcon, Tokyo, Japan) depicting corneal endothelial decompensation with central corneal edema, Descemet’s folds, and epithelial bullae.

Intraoperative complications included two cases of self-limited hyphema in both groups and a case of aqueous misdirection syndrome resolving with intravenous mannitol. Postoperative complications included one case of wound leak in the 45 µm device, treated with a contact lens. A dysesthetic bleb with secondary Dellen in the 63 µm group was noted, which underwent surgical revision, achieving a favorable postoperative outcome. A case of anterior segment syndrome (TASS), with IOP spike, Urretz–Zavalia syndrome, and corneal decompensation was reported in the 45 µm group. Eventually, the patient required Descemet stripping automated endothelial keratoplasty (DSAEK) and TBT, due to poor IOP control (Figure 4).

Postoperative hypotony (defined as an IOP < 6 mmHg) was more common in the 63 µm group (11 cases, 39.3%) than in the 45 µm group (8 cases, 28.6%), with no statistical differences (Pearson Chi-square, *p* = 0.397). Hypotony-associated complications (including choroidal detachment, hypotony keratopathy, and hypotony maculopathy) were significantly higher in the 63 µm group (Fisher’s exact test, *p* = 0.011). Four cases of serous choroidal detachment and a case of localized hemorrhagic choroidal associated with hypotonic keratopathy and hypotonic maculopathy, occurred in the 63 µm group. Meanwhile, only one case of serous choroidal detachment was noted in the 45 µm group. All these complications occurred in the first month, and most patients evolved favorably with conservative management, rest, and adjusted topical treatment (topical mydriatics every 8 h and topical phosphate dexamethasone every 2 h, with progressive tapering). In one patient with the XEN^®^ 63 µm implant, anterior chamber reformation with viscoelastic was performed in the anterior chamber due to sustained hypotony and serous choroidal detachment. There was no long-term visual acuity nor visual field loss secondary to hypotony-associated complications. Moreover, there was no statistical association between the use of bleb space modulators and postoperative hypotony (Pearson Chi-square, *p* = 0.916) nor hypotony-associated complications (Fisher’s exact test, *p* = 0.103). Additionally, postoperative cystoid macular edema developed in one patient after the 63 µm implant, resolving with one dose of sub-Tenon’s triamcinolone.

## 4. Discussion

To the best of our knowledge, this is the first study comparing the efficacy and safety of XEN^®^ 45 µm and XEN^®^ 63 µm with the AEO approach. Although Hussein IM et al. [15] reported a comparison of both devices, the surgical technique used was different as they employed an ab interno approach with both closed and open conjunctiva. Additionally, Fernandez-Garcia A et al. compared both devices using an AIC approach [16]. In our study, we observed that both devices achieved a significant reduction in intraocular pressure and the use of glaucoma medication. However, despite observing a trend toward a higher rate of both complete and qualified surgical success in the 63 µm group, these differences did not reach statistical significance. Likewise, postoperative IOP was lower, with lesser requirements of glaucoma medications in the 63 µm group, although these differences were not statistically significant. Regarding safety, although the incidence of hypotony was similar in both groups, a significantly higher rate of hypotony-related adverse events was observed in the XEN^®^ 63 µm group. Hypotony incidence was not associated with the use of bleb modulators. However, in our cohort of patients, no cases of hypotony required surgical revision, and no visual acuity loss secondary to hypotony was detected.

Fernández-García A et al. [16] compared the differences between both devices in the medium and long term, with a follow-up extended to 3 years. Although both devices demonstrated efficacy in IOP, no statistically significant differences were observed in the use of hypotensive eye drops or IOP, except at 12 months, where the XEN^®^ 45 µm group had lower IOP. Furthermore, the authors reported a good safety profile in both treatment groups, with no differences in the need for secondary needling. However, they did not report cases of hypotony or specify whether complications associated with this condition had occurred. On the other hand, Hussein IM et al. [15], who published the largest series comparing both devices, reported a significantly greater reduction in IOP and the number of hypotensive medications in the group treated with XEN^®^ 63 µm. Furthermore, they observed a significantly higher surgical failure rate in the XEN^®^ 45 µm group, although no significant differences were found regarding the need for secondary needling. Additionally, they reported a significantly higher rate of hypotony and associated complications in the 63 µm group, as in our study [15]. Considering the Poiseuille’s law, in the 63 µm device, the resistance to outflow is 3.8 times lower compared to the 45 µm implant. Therefore, this conveys a potentially higher IOP reduction and an increased risk of hypotony and associated complications, particularly in the early postoperative period, as described in our study. Indeed, particular concern should be addressed in high-myopic or aphakic patients, who are especially prone to developing hypotony-associated complications. Likewise, the diminished resistance increases the outflow, theoretically achieving greater IOP reduction and lower postoperative levels, as described by Hussein IM et al. [15].

However, in our study, although better long-term surgical survival was observed with lower mean IOP in the XEN^®^ 63 µm group, these differences were not statistically significant. The lack of statistical significance could be explained first by the sample size. Furthermore, the external approach with conjunctival opening and meticulous dissection of the conjunctiva and Tenon’s capsule may improve outflow through the device, which could attenuate the differences related to device diameter. The efficacy and safety of the AIC XEN^®^ implant, either standalone or combined with phacoemulsification, has been reported for both the 45 µm [17,18] and the 63 µm [19,20] devices. However, in recent years, off-label ab externo has gained popularity, as it may offer certain advantages compared to the ab interno technique [11,12,13,14]. The AEO approach allows for a thorough dissection of the conjunctiva and Tenon’s capsule, optimizing the visualization of the device, limiting outflow resistance, and preventing Tenon’s entanglement, which is associated with early surgical failure [21,22]. Bleb space modulators such as Healaflow^®^ or Ologen^®^ may be correctly placed thanks to direct visualization, and proper MMC application to the scleral bed may be ensured. Additionally, anterior chamber manipulation is limited in the AEO approach, with no need for viscoelastic or a corneal main incision if a standalone procedure is performed. In this article, we describe a modified version of the AEO procedure. Indeed, a 30G needle is used to create the scleral tract instead of using the 27G injector. This limits the peritubular leakage, the main factor associated with early hypotony, guaranteeing the long-term functionality of the implant and reducing the incidence of secondary needling. The 30G needle allows for an easier and more controlled approach compared to the use of the injector. The latter, as it has a retractable system, requires an entrance closer to the limbus and has not been designed for an open-conjunctiva approach. Although this technique implies a longer operating time, with the potential risk of device breakage, no intraoperative complications were reported.

This study has several limitations that should be addressed. First, the small sample size and retrospective design may limit the generalizability of the results obtained. Its retrospective and non-randomized design implies a significant risk of selection bias. The choice between XEN^®^ 45 µm and XEN^®^ 63 µm was not assigned randomly but rather determined by the surgeon’s clinical judgment and the implant availability, or individual patient characteristics that are not always systematically recorded. This variability may have generated groups with baseline differences not captured by the baseline variables, potentially affecting the observed results in terms of both efficacy and safety. Therefore, the results should be interpreted with caution, understanding that they reflect clinical experience in a real-world setting but without the control that would allow for robust causal inference. Although the surgical technique used was standardized, the interventions were performed by different surgeons, introducing potential procedural variability. However, we believe that this heterogeneity could more accurately reflect daily clinical practice, giving relevance to our findings in real world settings. Additionally, one of the challenges associated with MIBS and MIGS is the long-term surgical success. Although the short- and medium-term results are promising, it is crucial to evaluate the long-term efficacy of these techniques, especially in comparison with conventional filtering surgery, which has demonstrated sustained efficacy over time.

## 5. Conclusions

This study helps contribute to the limited evidence on the comparison between both devices using the AEO approach. Based on our findings and the preliminary data, we suggest that in patients with higher baseline IOP or severe glaucoma, the 63 µm device may be preferred over the 45 µm device. In cases with hypotony predisposing ophthalmological or systemic conditions, we believe the 45 µm device should be prioritized to ensure a safer postoperative course. Prospective studies and randomized clinical trials with extended follow-up and a larger sample size are necessary to confirm these findings.

## Figures and Tables

**Table 1:** Demographic and baseline characteristics.

Characteristics	XEN 63 µm	XEN 45 µm	*p*-Value
Eyes (*n*) Eye laterality (right)	28 13 (46.4%)	28 16 (57.1%)	0.431
Age (y), mean (SD) [max; min]	70.1 ± 18.3 [33; 89]	69.9 ± 15.9 [61; 88]	0.482
Female sex (%)	14 (50%)	13 (54.5%)	0.603
Preoperative mean BCVA (logMar) [IQR]	0.2 [0.05; 0.35]	0.3 [0.15; 0.45]	0.684
Preoperative mean IOP (mmHg) (SD)	23.9 ± 2.5	23.1 ± 2.2	0.736
Glaucoma type (%) *Primary open angle glaucoma* *Pseudoexfoliative* *Juvenile* *Uveitic* *Others*	18 (64.3%) 3 (10.7%) 2 (7.1%) 2 (7.1%) 3 (10.7%)	22 (78.6%) 3 (10.7%) 0 (0.0%) 1 (3.6%) 2 (7.1%)	0.633
Glaucoma staging (%) *Mild * *Moderate* *Severe*	7 (25%) 6 (21.5%) 15 (53.5%)	7 (25%) 5 (17.9%) 16 (57.1%)	0.913
Mean number of medications (SD)	2.6 ± 0.9	2.5 ± 1.1	0.716
Combined surgery (%)	9 (32.1%)	11 (39.3%)	0.640
Visual field mean (dB) deviation [IQR]	−7.1 [−14.4;−4.5]	−6.8 [−13.8;−3.9]	0.651
Mean axial length (mm) (SD)	23.1 ± 2.0	22.7 ± 1.5	0.554
Previous SLT (%)	2 (7.1%)	2 (7.1%)	1
Previous glaucoma surgeries (%)	3 (10.7%)	2 (7.1%)	0.665
Other procedures (%) *Cataract surgery* *LASIK* *Pars plana vitrectomy* *Keratoplasty*	9 (32.1%) 1 (3.6%) 2 (7.1%) 0 (0.0%)	14 (50.0%) 1 (3.6%) 2 (7.1%) 1 (3.6%)	0.182
Use of intraoperative MMC (%)	28 (100%)	28 (100%)	
Use of bleb space modulators (%)	18 Healaflow^®^ (64.3%)	16 Ologen^®^ (57.1%)	0.435

BCVA: best-corrected visual acuity; IOP: intraocular pressure; logMAR: logarithm of the minimum angle of resolution; MD: mean deviation (dB); LASIK: *laser* in situ *keratomileusis*; MMC: mitomycin C.

## Data Availability

The datasets used and/or analyzed during the current study are available from the corresponding author on reasonable request.

## Abbreviations

AEO	Ab Externo with open conjunctiva
AIC	Ab Interno with closed conjunctiva
AS-OCT	Anterior segment optical coherence tomography
BSS	Balanced salt solution
BVCA	Best-corrected visual acuity
CME	Cystoid macular edema
DB	Decibels
DSAEK	Descemet stripping automated endothelial keratoplasty
GATT	Gonioscopy-assisted transluminal trabeculotomy
HR	Hazard ratio
IOP	Intraocular pressure
IQR	Interquartile range
LASIK	Laser-assisted in situ keratomileusis
logMAR	Logarithm of the minimum angle of resolution
MD	Mean deviation
MIBS	Minimally invasive bleb surgery
MIGS	Minimally invasive glaucoma surgery
MMC	Mitomycin C
NPDS	Non-penetrating deep sclerectomy
OCT	Optical coherence tomography
ONH-OCT	Optical coherence tomography of the optic nerve head
SD	Standard deviation
SLT	Selective laser trabeculoplasty
SPSS	Statistical Package for the Social Sciences
TASS	Toxic anterior segment syndrome
TBT	Trabeculectomy
VF	Visual field
